# Prevalence and risk factors of intestinal parasites among children under two years of age in a rural area of Rutsiro district, Rwanda – a cross-sectional study

**DOI:** 10.11604/pamj.2019.32.11.15949

**Published:** 2019-01-07

**Authors:** Eric Butera, Assumpta Mukabutera, Etienne Nsereko, Cyprien Munyanshongore, Nadine Rujeni, Ivan Emile Mwikarago, Patricia Jean Moreland, Manasse Nzayirambaho Manasse

**Affiliations:** 1 University of Rwanda, College of Medicine and Health Sciences, School of Public Health, Rwanda; 2 University of Rwanda, College of Medicine and Health Sciences, School of Health Sciences, Rwanda; 3 Rwanda Ministry of Health, Rwanda Biomedical Center, National Reference Laboratory, Rwanda; 4 Emory University, Nell Hodgson Woodruff School of Nursing, USA

**Keywords:** Water treatment, sanitation facility, ascaris, Sub-Saharan Africa

## Abstract

**Introduction:**

This study aimed to assess the prevalence and associated risk factors of intestinal parasite infections among children less than two years of age in Rutsiro, Rwanda.

**Methods:**

A cross-sectional parasitological survey was conducted in Rutsiro in June 2016. Fresh stool samples were collected from 353 children and examined using microscopy to detect parasite. A questionnaire was administered to collect data on hygiene, sanitation, socio-demographic and economic characteristics.

**Results:**

Approximately one in two children (44.8%) were found to be infected with at least one intestinal parasite. Ascaris (28.5%) was the most prevalent infection followed by Entamoeba histolytica (25.95%) and Giardia lamblia (19.6%). Infection with more than one pathogen was noted e.g. presence of Ascaris and yeasts (8.9%), and amoeba with Trichocephale (4.4%), respectively. Children from non-farming families were less likely to be at risk of intestinal parasite infections (AOR = 0.41, p = 0.028) compared to children from farming families. Children from households with access to treated drinking water were less likely to contract intestinal parasite infections (AOR = 0.44, p = 0.021) compared with those who used untreated water. Children from families with improved sources of water were twice as likely to be diagnosed with intestinal parasitoses compared to those who did not. We postulate that the majority of families (50.1%) who have access to improved water sources do not treat water before consumption.

**Conclusion:**

The high prevalence of intestinal parasitoses in children warrants strict control measures for improved sanitation, while treatment of drinking water should be considered.

## Introduction

Intestinal parasite infections significantly affect public health in developing countries [1, 2], and they are responsible for major morbidity and mortality throughout the world [3]. The reasons for the developing world being disproportionately affected includes over-crowding, poor environmental sanitation and hygienic practices [4]. Worldwide, approximately 3.5 billion people and 450 million people suffer from these infections, with children most affected and more likely to present with clinical symptoms [5, 6]. Children may be infected with one or more of these intestinal parasites, affecting the child's immune system and increasing susceptibility to risk of other diseases. While mortality from intestinal parasite protozoan infections is relatively unusual, morbidity and indirect effects have significant consequences on health. These include gastrointestinal disorders, diarrhea, dysentery, vomiting, lack of appetite, hematuria, abdominal distension and mentally related health disorders, which can contribute to mortality [7].

Poor sanitation and inadequate hygiene [4, 8] have been identified as significant contributors to intestinal parasite infections and these indicators constitute a critical problem for Rwanda, as they put Rwandan children at risk of intestinal parasitoses. As reported in the Rwanda demographic and health survey (RDHS, 2014/2015), hygiene practice is still a problem. For example, the RDHS found that only 12% of the population have access to hand washing facilities and only 3% of those resident in Rutsiro district have access [9]. According to clinical reports at the local Health Center, intestinal parasite infections are listed among the top reasons why people visit health facilities in Rwanda. Confirming the spread and incidence/prevalence of intestinal parasite infection is very important for the design and implementation of appropriate prevention and control measures to mitigate against child morbidity. However, little is known on the prevalence and risk factors associated with intestinal parasitic infections amongst those who live in rural Rwanda. Therefore, the objective of the present study was to assess the prevalence of intestinal parasitic infections among children under two years of age in rural Rwanda, in order to inform researchers and decision-makers on measures to improve children's health and nutritional status.

## Methods

**Study area and population:** A community cross-sectional study was conducted, in June 2016, among children under two years of age in Rutsiro district, Rwanda. Rutsiro is one of seven districts of Rwanda (Karongi, Nyabihu, Rubavu, Rusizi, Ngororero, Nyamasheke and Rutsiro) located in the western province, the population of Rutsiro district is estimated to be approximately 324,654 habitants, with a density of 280/km^2^ and a total surface area of 1159 km^2^. Agriculture is the main source of income for the district.

**Selection of study subjects:** Samples of 353 children less than two years of age were randomly selected from all the households in the district. Firstly, we randomly selected 13 sectors in Rutsiro district, then from the selected sectors, we chose cells, which included designated villages termed “Imidugudu”. Lastly, households were also selected using a systematic random sampling method for households chosen by village leaders in collaboration with community health workers.

**Stool sample collection and examination:** From each child, fresh stool samples were collected using appropriate containers. They were kept in accordance with national laboratory standards and then examined using a direct smear process by biomedical lab technicians in the field. Descriptive statistics were used to explain our sample according to socio-economic and demographic characteristics. To identify the risk factors associated with intestinal parasitic infection, a multivariable logistic regression was used. All associated factors in bivariate analysis including environmental factors were entered into the full model and stepwise regression was used to obtain the final model. Odds ratios (OR) and their 95% confidence intervals (CI) were presented. Statistical significance was fixed at p ≤ 0.05. Data analysis was performed using Stata software, version 13 (StataCorp LP, College Station, TX, USA).

**Ethical considerations:** The study was reviewed and approved by the ethical committee of University of Rwanda, College of Medicine and Health Sciences. In addition to this IRB approval, we obtained permission to collect data from the administrative authority (District and Sector). Parents signed an informed consent form prior to the data collection exercise for anonymity and there was no identification of the interviewees available to researchers. The ethical considerations were addressed by treating the infected individuals using standard anti-microbial drugs under the supervision of a local nurse.

## Results

A total of 353 children under the age of two were selected for the study (Table 1). Microscopic stool sample examination revealed that approximately one in two children (n = 158, 44.8%) out of the 353 children surveyed were found to be infected with at least one intestinal parasite. The prevalence of Ascaris (28.5%) was the highest followed by amoeba (Entamoeba histolytica or coli) (25.95%), Giardia lamblia (19.6%), and yeasts (10.1%). Infection with more than one intestinal parasite such as Ascaris and yeast (8.86%) and amoeba with Trichocephale (4.43%) was noted, respectively. Other intestinal parasites detected such as Necator americanus and Trichomonas hominis were identified at less than one percent (0.63%) prevalence (Table 2). Of the 353 participants, half reported drinking untreated water (n = 177, 50.1%), 44% used drinking water from an unimproved source, and 31.4% stayed with livestock in the household (Table 2). The factors associated with having any intestinal parasites included farming, water treatment, and water source (Table 3). Children from non-farming families were less likely to be diagnosed with intestinal parasites (AOR = 0.41, p = 0.028) compared with children of farming families. Furthermore, children from households with access to treated drinking water were less likely to be diagnosed with intestinal parasite infections (AOR = 0.44, p = 0.021), compared to those who drank untreated water (Table 4). Children from families with improved water sources were twice as likely to be diagnosed with intestinal parasite infections compared to those who did not have access to improved water sources. We postulate that this is because the majority of families (50.1%) who have access to improved water sources do not treat their water before use (Table 2).

**Table 1:** Socio-demographic characteristics of participants, Rutsiro, Rwanda, 2016 (n = 378)

Variables		Frequency	%
**Child age in months**			
	<12 Months	91	25.78
	12-17 Months	118	33.43
	18-23 Months	135	38.24
	Missing	9	2.55
**Child sex**			
	Male	151	42.78
	Female	190	53.82
	Missing	12	3.4
**Mother age**			
	16-25yrs	80	22.66
	26-30yrs	86	24.36
	31-35year+	94	26.63
	36+year	92	26.06
	Missing	1	0.28
**Marital status**			
	Married	296	83.85
	Single mother	57	16.15
**Possession of livestock**			
	No	200	56.66
	Yes	153	43.34
**Occupation**			
	Not farmers	129	36.65
	Farmers	223	63.35
**Mother literacy**			
	Yes	210	59.66
	No	142	40.34
**wealth category**			
	Category 1	275	77.9
	Category 2	55	15.58
	Category 3	22	6.23
	Missing	1	0.28
**Total**		353	100

**Table 2:** Prevalence of intestinal parasites in children under two years from Rutsiro district

Variables	Frequency	Percent
**STH Infection**		
No	185	53.94
Yes	158	46.06
**Intestinal parasite species**		
Ascaris	45	28.48
Entamoeba Histo. or coli	41	25.95
Giardia lamblia f.veg	31	19.62
yeasts	16	10.13
Ascaris, K. Giardi, E. coli	14	8.86
Tricocephales	7	4.43
Trichiris trichiura	2	1.27
Necator americanus	1	0.63
Trichomonas hominis	1	0.63
**Child has Intestinal parasite**		
No	185	53.94
Yes	158	46.06
**Livestock at home**		
No	200	56.66
Yes	153	43.34
**Drinking water treatment**		
No	177	50.14
Yes	176	49.86
**Water source**		
Unimproved	156	44.19
Improved	197	55.81
**Distance from kraal**		
<5m	130	80.25
>5m	32	19.75
**Stay with livestock in household**		
Yes	80	31.37
No	175	68.63
**Child stool disposal**		
Unimproved	21	5.97
Improved	331	94.03
**Toilet quality**		
Unimproved	334	94.62
Improved	19	5.38
**Total**	353	100

**Table 3:** Association between intestinal parasite infections, socio-demographic and economic characteristics, and WASH indicators

Variables	N (frequency)	No intestinal parasites	Child had intestinal parasites	P value
**Child sex**				0.281
Male	145	74(51.03)	71(48.97)	
Female	186	106(56.99)	80(43.01)	
**Child age**				0.253
<12 Months	87	49(56.32)	38(43.68)	
12-17 Months	118	69(58.47)	49(41.53)	
18-23 Months	130	63(48.46)	67(51.54)	
**Wealth categories**				0.874
Category 1	267	146(54.68)	121(45.32)	
Category 2	55	28(50.91)	27(49.09)	
Category 3	20	11(55)	945	
**Possession of livestock**				0.059
No	194	96(49.48)	98(50.52)	
Yes	149	89(59.73)	60(40.27)	
**Farming**				<0.001
Not farmers	125	51(40.8)	74(59.2)	
Farmers	217	134	83(38.25)	
**Literacy**				0.13
Yes	206	104(50.49)	102(49.51)	
No	136	80(58.82)	56(41.18)	
Livestock				0.059
No	194	96(49.48)	98(50.52)	
Yes	149	89(59.73)	60(40.27)	
**Drinking water treatment**				0.038
Water not treated	174	87(50)	87(50)	
water treated	169	98(57.99)	71(42.01)	
**Drinking water source**				0.052
Unimproved source	153	91(59.48)	62(40.52)	
Improved source	190	94(49.47)	96(50.53)	
**Distance from source of water**				0.429
<5m	126	73(57.94)	53(42.06)	
>5m	32	21(65.63)	11(34.38)	
**Live with livestock in house**				0.458
Yes	78	47(60.26)	31(39.74)	
No	172	95(55.23)	77(44.77)	
**Child stool disposal**				0.539
Bad disposal	21	10(47.62)	11(52.38)	
Good disposal	321	175(54.52)	146(45.48)	
**Toilet quality**				0.907
Unimproved toilet	324	175(54.01)	149(45.99)	
Improved toilet	19	10(52.63)	9(47.37)	

**Table 4:** Factors associated with STH among children under two years, Rutsiro district, Rwanda, 2016 (n = 353)

Variables	Full Model			Reduced model		
	COR	P value	COR 95% CI	A OR	P value	AOR 95%CI
**Farming**						
Not Farmers	1.00					
Farmers	0.38	0.035	0.16-0.93	0.41	0.028	0.18-0.91
**Literacy**						
Yes	1.00					
No	0.46	0.061	0.20-1.04	0.48	0.063	0.22-1.04
**Livestock**						
No	1.00					
Yes	1.42	0.594	0.39-5.12	-	-	-
**Drinking water treatment**						
Water not treated	1.00					
Water treated	0.42	0.018	0.21-0.86	0.44	0.021	0.22-0.88
**Drinking water source**						
Unimproved source	1.00					
Improved source	2.12	0.04	1.03-4.36	2.05	0.045	1.02-4.15
**Distance from kraal**						
<5m	1.00					
>5m	0.85	0.74	0.33-2.21	-	-	-
**Live with livestock in house**						
Yes	1.00					
No	1.39	0.392	0.66-2.94	-	-	-
**Child stool disposal**						
Bad disposal	1.00					
Good disposal	0.09	0.062	0.01-1.13	0.10	0.066	0.01-1.17
**Toilet quality**						
Unimproved toilet	1.00					
Improved toilet	0.66	0.531	0.18-2.45	-	-	-
**Constant**	**12.52**	**0.081**	**0.73-21.48**	**16.17**	**0.038**	**1.16-22.51**

**Abbreviations:** COR, Crude Odds Ratio; AOR, Adjusted Odds Ratio; CI, Confidence Intervals

## Discussion

Confirming the spread and level of intestinal parasite infections is very important in the design and implementation of appropriate prevention and control measures for child morbidity. This study was conducted to determine the prevalence of intestinal parasite infections and associated risk factors in children less than two years of age in Rutsiro district, a rural part of Rwanda. Almost half of the 353 children surveyed for intestinal parasite infections were found to be infected with at least one intestinal parasite. Ascaris was the most commonly identified with approximately one in three children infected with Ascaris. Furthermore, almost one in ten children were dually infected with Ascaris combined with other parasites and fungal organisms (amoeba and yeasts). In comparison to other intestinal parasites, the prevalence of Ascaris was comparable to the prevalence of Giardia duodenalis in a study assessing intestinal parasites among children [10]. This study of children in Rutsiro District indicates that apart from Ascaris, the prevalence of Giardia was higher than other intestinal parasites detected. Furthermore, the risk factors associated with intestinal parasite infection included farming occupation, drinking untreated water, and non-improved water sources. Children of farming parents were more likely to be infected with intestinal parasites compared to children of parents who did not farm. These parents were more likely to have an occupation which involved casual labour, food vending, or small commerce [11]. Although the prevalence of hookworm was high in the study subjects, data on the number of eggs per infected individual suggested that infection was at a low intensity.

Children from households with access to treated drinking water were less likely to have intestinal parasite infections, compared to those who drank untreated water. Surprisingly, we discovered that children from families with access to improved source of water were twice as likely to be diagnosed with an intestinal parasite infection compared to those from families without access to improved sources of water. It is possible that a significant proportion of mothers who receive water from trusted and improved sources of water do not treat the water before drinking it, a practice that would be mirrored by their children. As a result of the high prevalence of hookworm infection in the study subjects, an attempt was made to assess the association between hookworm infection and haematocrit values in the study subjects. In agreement with the previous report from southern Rwanda [11], the present study revealed there was no significant association between low haematocrit values and hookworm infection. In contrast, other studies have shown a strong association between low haematocrit values and hookworm infection [12, 13]. This could be explained by low intensity of hookworm infection, nutritional status of the study subjects or due to differences in the species of hookworm [14].

## Conclusion

In conclusion, the study showed that intestinal parasites were prevalent in children less than two years of age in Rutsiro district, a rural and remote area of Rwanda. This calls for control measures such as community mobilization regarding water treatment, sanitation improvement, and maintaining regular adherence to de-worming programs for children.

### What is known about this topic

- Consequences of intestinal parasite infections on health;
- Contributors to intestinal parasite infections globally.

### What this study adds

- The prevalence and risk factors associated with intestinal parasitic infections amongst those who live in rural Rwanda;
- Most prevalent intestinal parasitic infections in rural Rwanda.

## Competing interests

The authors declare no competing interests.
